# Developing and validating a Japanese version of the Plymouth Sensory Imagery Questionnaire

**DOI:** 10.3389/fpsyg.2023.1166543

**Published:** 2023-06-23

**Authors:** Jimpei Hitsuwari, Michio Nomura

**Affiliations:** Department of Education, Kyoto University, Kyoto City, Japan

**Keywords:** multi-sensory imagery, mental imagery, scale development, individual differences, cultural comparison, assessment, mindfulness

## Abstract

Mental imagery refers to the representation of stimuli that are not physically present and has long been a subject of interest in psychology. However, most research on mental imagery has been limited to visual images, with other types of imagery, such as sound and smell, receiving little attention. A possible reason for this is the lack of appropriate scales to measure the vividness of multisensory imagery. The Plymouth Sensory Imagery Scale (Psi-Q) has been developed to address this issue and has been used in several studies to measure the vividness of seven imageries: vision, sound, smell, taste, touch, body, and feeling. In this study of 400 participants in Japan, the Psi-Q was translated into Japanese and tested for reliability and validity. The results showed good internal reliability and retest reliability and moderate to high correlations with other measures of construct validity, including mindfulness, Big Five, and life satisfaction. Additionally, there is no significant difference in total Psi-Q scores between the Japanese and British samples, although some differences are found in individual sensory imagery abilities. This study provides valuable insights into multisensory mental imagery, and it is expected that research dealing simultaneously with the responses of multisensory modalities will further accumulate.

## 1. Introduction

Mental imagery is defined as “an internal representation of stimuli that are not physically present (Shaw, 2008, p. 175)” and has been a central theme in psychology over the past several decades (Pylyshyn, 1973; Pearson, 2019). The range of topics utilizing mental imagery includes physiology, perception, learning, memory, and exercise (Hatakeyama, 2019), and applied research has been conducted in areas such as marketing and medicine (e.g., Pearson et al., 2015; Zaleskiewicz et al., 2020). However, most of these studies regarding mental imagery have been limited to vision; other mental imagery, such as sound and smell, have been studied less actively (Andrade et al., 2014). This is contrary to the growing interest in the topic of crossmodal correspondence, which attempts to examine the interaction of multiple sensory perceptions rather than just one (Uno and Yokosawa, 2022). One possible reason for this is that there is a lack of a scale with sufficient validity and reliability available to measure the vividness of multisensory imagery simultaneously. In reality, some scales such as the Questionnaire upon Mental Imagery (QMI; Betts, 1909; Sheehan, 1967 for short version) and the Survey of Mental Imagery (SMI; Switras, 1978; Grebot, 2003 for French version), which can measure multisensory imagery ability, have been developed, but these measures are problematic in three aspects. First, the scales were not created through adequate psychometric testing (McAvinue and Robertson, 2007; Andrade et al., 2014). For example, the SMI does not examine correlations with existing scales to determine construct validity, and there are no reports of test–retest reliability. Second, the QMI and SMI are long scales with 150 and 86 items, respectively, which has the disadvantage of placing a high burden on respondents. Third, these scales have been in development for a long time, and the wordings of some items are not suitable today. For example, the QMI uses items that reference the whistle of a locomotive (sound), velvet (touch), and smoke from a train (smell), which are no longer common experiences. Based on these observations, the Plymouth Sensory Imagery Scale (Psi-Q; Andrade et al., 2014) was developed to overcome these problems and simultaneously measure the vividness of seven modality imageries: vision, sound, smell, taste, touch, body sensation, and feeling. Andrade et al. (2014) sampled extensively across multisensory modalities, reviewing the QMI and the Vividness of Visual Imagery Questionnaire (VVIQ; Marks, 1973). Consequently, two items were retained and eight items were rephrased from the short version of the QMI, and 25 new items were added. The scale was developed through three experiments (*N* = 854). This has demonstrated construct validity (*r* = 0.18–0.40 with the Spontaneous Use of Imagery Scale (SUIS); Reisberg et al., 2003), internal reliability (*α* = 0.93–0.96 for the Psi-Q; *α* = 0.80–0.97 for the subscales), and retest reliability (*r* = 0.43–0.84). Since then, the Psi-Q has been translated worldwide, including a Spanish version (Pérez-Fabello and Campos, 2020) and a Dutch version (Woelk et al., 2022), and has become a leading scale for measuring multisensory imagery ability. The Spanish version showed a seven-factor structure as in the original version, despite having removed four items, with adequate to high internal consistency (*α* = 0.92 for the Psi-Q; *α* = 0.68–0.77 for the subscales) and correlation with the QMI (*r* = 0.40–0.56), confirming its construct validity. The Dutch version showed a seven-factor structure, similar to the original version, with adequate to good internal consistency (*α* = 0.94–0.96 for Psi-Q; *α* = 0.76–0.88 for subscales) and test–retest reliability (*r* = 0.83 for Psi-Q; *r* = 0.67–0.75 for subscales), and construct validity (*r* = 0.32 with SUIS) using two surveys of students and a general sample. Translating and developing the Psi-Q in Japan will ensure the measurement of multisensory imagery ability simultaneously and confirm that the same factor structure can be found across cultures.

The Psi-Q has been employed in a variety of surveys and experiments (e.g., Kharlas and Frewen, 2016; Clark et al., 2020; Koivisto and Grassini, 2022). Kharlas and Frewen (2016) have examined a trait mindfulness scale’s association with the Psi-Q and found moderate positive correlations between its five subscales and each of the Psi-Q’s sensory imagery abilities. In particular, the “Observe” factor of the trait mindfulness scale was significantly correlated with mental imagery in all seven sensory organs (*r* = 0.23–0.47, *ps* < 0.01), while only bodily and feeling imagery was not correlated with “Act with awareness” (*r* = 0.06–0.18, *ps* > 0.05), suggesting that there were differences across sensory organs. In addition, the Psi-Q has been used in behavioral experiments; for example, Koivisto and Grassini (2022) have shown that images of nature (vs. urban environment and architecture) are associated with positive affect and relaxation. They further have found that visual imagery ability in the Psi-Q predicted the vividness of imagery most successfully (*B* = 0.34, *p* < 001), while bodily imagery ability predicted relaxation most successfully (*B* = 26, *p* = 0.002). Thus, it is important to simultaneously measure individual differences in multisensory imagery ability and identify commonalities and differences among sensory organs. In this study, in addition to mindfulness, we further aim to deepen our understanding of multisensory mental imagery by examining its relevance to other scales, such as the Big Five and Satisfaction with Life Scale, because these measures have not yet been examined in relation to multisensory imagery abilities but have been shown to be associated with single modalities such as visual imagery (e.g., Wilson et al., 2018; Budnik-Przybylska et al., 2023). We hypothesized, with reference to these previous studies, that multisensory imagery ability would correlate positively with mindfulness, negatively with neuroticism, positively with openness and extraversion, and positively with life satisfaction.

Scales measuring the individual differences in mental imagery have developed for each modality rather than multiple modalities simultaneously. For example, the Vividness of Visual Imagery Questionnaire (VVIQ; Marks, 1973), Vividness of Olfactory Imagery Questionnaire (VOIQ; Gilbert et al., 1998), and Auditory Imagery Questionnaire (AIQ; Hishitani, 2009), which measure visual, olfactory, and auditory imagery abilities, respectively, have been developed. Japanese versions of these scales likewise exist and have been widely used (Hishitani, 2005; Hishitani, 2011; Yamamoto et al., 2018). However, there is a growing need to simultaneously measure multisensory mental imagery abilities for use with other scales and behavioral experiments; therefore, it is important to develop a Japanese version of the Psi-Q.

To our knowledge there are no studies examining the cultural differences in multisensory imagery ability. While not comparing countries, Talamini et al. (2022) has examined musicians’ and non-musicians’ questionnaire responses regarding visual and auditory imagery abilities and found that musicians had higher auditory imagery abilities than non-musicians, however, there was no difference in visual imagery abilities. The results suggest that mental imagery is influenced by culture and environment and that this influence differs from one sensory system to another. These differences are not only in the concepts that have been treated in psychology, such as the cultural self (Markus and Kitayama, 1991) and genotypes associated with depression (Chiao and Blizinsky, 2010), but also in the customs and food culture. Therefore, it is vital to exploratively examine the cultural differences of multisensory imagery.

### 1.1. Aims

In this study, we first translated the original version of the Psi-Q (Andrade et al., 2014) to develop a Japanese version, and then examined its validity and reliability. To examine construct validity, we measured existing imagery ability scales, such as the VOIQ, and examined relationships with individual differences in the Big Five, trait mindfulness, and life satisfaction. Finally, an exploratory examination of cultural differences in multisensory imagery ability between the United Kingdom and Japan was conducted using original data (Andrade et al., 2014). This study can inspire research on cultural comparisons of multisensory imagery abilities which have not yet been directly compared, despite the repeated implication that they are influenced by culture and environment.

## 2. Methods

This study was approved by the Ethics Committee of Kyoto University (CPE-496). All data and scripts are available online.^1^

### 2.1. Participants

Four hundred people were recruited through the Japanese crowdsourcing platform CrowdWorks.^2^ Of these, 389 (*M* = 40.72, *SD* = 10.73; 155 men, 234 women) were analyzed, excluding those with extremely short response times and those who missed the attention check. To examine retest reliability, the same participants were invited to respond again 1 week later. Data from 344 participants (*M* = 40.85, *SD* = 10.65; 137 men, 207 women), whose ID matched their first completed questionnaire, were used for the retest reliability analysis.

### 2.2. Materials

#### 2.2.1. The Plymouth sensory imagery scale (Psi-Q)

After obtaining permission from the original authors, the first author, a fluent and native Japanese speaker, translated the Psi-Q into Japanese (Andrade et al., 2014). All items used in the original version of the Psi-Q were not changed to consider cultural influences because they all are familiar to Japanese. For the Japanese version, we conducted three preliminary surveys with a sample size of 100–150 participants and made minor revisions to the items. Back-translation was then performed using a translation service (NAI Inc.^3^). The original author was then asked to confirm whether there were any differences in meaning or intent from the original version. The original version of the scale consists of seven subscales, five items each, for “vision,” “sound,” “smell,” “taste,” “touch,” “body,” and “feeling,” for a total of 35 items. A shortened version, with three items each for a total of 21 items, has also been developed. For example, in the case of “sense of smell,” the participants are asked to respond to the question, “Imagine the smell of a rose,” using a 7-point scale from 1, “I cannot imagine it at all,” to 7, “It is as vivid as if it were right in front of my eyes.” The completed Japanese version of Psi-Q is described in Table 1.

**Table 1:** Factor loadings for each item in the factor analysis with a six-factor structure (only items with factor loadings of 0.4 or higher are shown).

	English	Japanese	*M*	SD	Vision	Sound	Smell	Taste	Touch	Body
Imagine the appearance of…										
1	*a bonfire	焚き火	5.67	1.19	0.75					
2	*a sunset	夕焼け	5.84	1.08	0.69					
3	*a cat climbing a tree	木に登る猫	5.35	1.39	0.52					
4	a friend you know well	よく知っている友人	5.74	1.26	0.51					
5	the front door of your house	自宅の玄関扉	6.16	1.02						
Imagine the sound of…										
6	*the sound of a car horn	車のクラクションの音	5.81	1.12		0.70				
7	*hands clapping in applause	割れんばかりの拍手	5.58	1.24		0.45				
8	*an ambulance siren.	救急車のサイレン	6.06	1.02		0.74				
9	the sound of children playing	子どもの遊ぶ声	5.71	1.21		0.57				
10	the mewing of a cat	猫の鳴き声	5.87	1.13		0.57				
Imagine the smell of…										
11	*newly cut grass	刈りたての草	4.66	1.55						
12	*burning wood	燃えている木	3.93	1.65			0.64			
13	*a rose	バラの花	4.21	1.83			0.49			
14	fresh paint	塗りたてのペンキ	4.43	1.55			0.52			
15	a stuffy room	むっとする部屋	4.27	1.53						
Imagine the taste of…										
16	*black pepper	ブラックペッパー	5.09	1.52				0.68		
17	*lemon	レモン	5.87	1.13				0.57		
18	*mustard	マスタード	4.88	1.50				0.74		
19	toothpaste	歯磨き粉	5.88	1.16				0.51		
20	sea water	海水	4.63	1.59						
Imagine touching…										
21	*fur	ふわふわとした毛皮	5.10	1.50					0.52	
22	*warm sand	暖かさをもった砂	4.83	1.50					0.64	
23	*a soft towel	柔らかいタオル	5.89	1.05						
24	icy water	氷水	5.84	1.13					0.45	
25	the point of a pin	ピンの先	4.82	1.65					0.53	
Imagine the bodily sensation of…										
26	*relaxing in a warm bath	温かいお風呂につかってリラックスする	5.98	1.09						0.68
27	*walking briskly in the cold	真冬に外で足早に歩く	4.86	1.53						0.46
28	*jumping into a swimming pool	プールの水面に飛び込む	4.64	1.57						0.48
29	having a sore throat	のどが痛む	5.59	1.21						0.55
30	threading a needle	針に糸を通す	5.50	1.26						0.50

M, mean; SD, standard deviation. * Indicates items used in the short version.

#### 2.2.2. The vividness of olfactory imagery questionnaire (VOIQ)

The VOIQ is a 14-item scale measuring olfactory imagery ability (Gilbert et al., 1998; Yamamoto et al., 2018). The responses are provided using a 5-point scale, ranging from 1, “I cannot smell anything at all, I just know that I am thinking about the smell that I am told,” to 5, “I can smell the smell completely clearly, as if I am smelling a real object.”

#### 2.2.3. The auditory imagery questionnaire (AIQ)

The AIQ is a 12-item scale measuring auditory imagery ability (Hishitani, 2009), using a 5-point scale ranging from 1, “I have no image at all, I just ‘know’ that I am thinking about what I am told,” to 5, “It is completely clear, like I am hearing a real thing.”

#### 2.2.4. Short version of the Japanese Big Five Scale

This shortened version of the Big Five Scale is a 29-item scale measuring the Big Five of extraversion, agreeableness, neuroticism, openness, and conscientiousness (Namikawa et al., 2012). The responses to the personality adjectives are provided on a 7-point scale ranging from 1, “not at all true,” to 7, “very true.” Although no studies have examined the relationship between multisensory mental imagery ability and Big Five personality, visual imagery ability and Big Five extraversion have been found to be positively related (McDougall and Pfeifer, 2012), and thus construct validity can be examined. In addition, we also examine the correlations between other personalities and mental imagery abilities in an exploratory manner.

#### 2.2.5. Short version of the five facet mindfulness questionnaire (FFMQ)

The shortened version of the Five Facet Mindfulness Questionnaire is a 24-item scale consisting of five factors (Observing, Non-reactivity, Non-judging, Describing, and Acting with awareness) measuring trait mindfulness (Takahashi et al., 2022). A 5-point scale ranged from 1, “not at all true,” to 5, “always true.” It has shown a moderate correlation with the Psi-Q (Kharlas and Frewen, 2016) and serves as an index for examining construct validity.

#### 2.2.6. The satisfaction with life scale (SWLS)

The Satisfaction With Life Scale is a one-factor, five-item scale measuring life satisfaction (Diener et al., 1985; Sumino, 1994). A 7-point scale ranging from 1, “not at all agree,” to 7, “very much agree,” is used to answer the questions. To our knowledge, there are no studies that examine the correlation between multi-sensory mental imagery ability and life satisfaction. However, well-being and mental imagery are related, as people with higher depression experience more negative imagery (Holmes et al., 2016); therefore, it will be interesting to look at the relationship between other-sensory imagery ability and life satisfaction in this study.

### 2.3. Procedure

The participants, recruited through Crowdworks, completed a web-based questionnaire created by Qualtrics. The participants were briefed on the survey and signed an informed consent form. They then began with responding to the Psi-Q, followed by the other five scales, which were presented in a randomized order. The items were also presented in a randomized order. The survey took approximately 10 min to complete. One week later, the same participants responded to the Psi-Q as described above.

### 2.4. Data analysis

First, using the *fa* function of the *psych* package (Revelle, 2022) in R (ver. 4.2.2; R Core Team, 2022), an exploratory factor analysis was conducted. A confirmatory factor analysis as well as a comparison of the models was then conducted using the *cfa* function from the *lavaan* package (Rosseel, 2012). Based on previous research (Yong and Pearce, 2013), we set a cutoff criterion of 0.4. To compare the model fit, we used chi square (χ^2^), goodness of fit index (GFI), adjusted GFI (AGFI), normed fit index (NFI), comparative fit index (CFI), root mean square error of approximation (RMSEA), and Akaike’s information criterion (AIC). To examine internal reliability, we used the *alpha* function from the *psych* package, and retest reliability was confirmed by calculating correlations with data obtained from the same sample 1 week later. For the British sample, we used data from Study 2 of Andrade et al. (2014), using the *t.test* function and the *mes* function from the *compute.es* package (Del Re, 2013) for effect size calculations.

## 3. Results

### 3.1. Factor structure

First, exploratory factor analysis was conducted to examine the eight-factor structure indicated by the scree plot, which resulted in only two items loading on “body” and two “feeling” factors (Supplementary Table S1). Alternatively, when the factor analysis was conducted assuming a six-factor structure, excluding the “feeling” factor from the original version, all factors loaded three or more items, resulting in a cohesive result (Table 1 and Supplementary Table S2 showing all factor loads).

As is clear from the model fit indices such as GFI and AIC in Table 2, the six-factor structure excluding the “feeling” factor is a better model than the original seven-factor structure.

**Table 2:** Comparison of the goodness of fit for each model.

	χ^2^	df	*P*-value	GFI	AGFI	NFI	CFI	RMSEA	AIC
7 factors, 35 items	1245.44	539.00	0.00	0.84	0.81	0.81	0.88	0.06	41290.04
6 factors, 30 items	806.49	390.00	0.00	0.87	0.85	0.85	0.92	0.05	35104.72
6 factors, 25 items	530.35	260.00	0.00	0.90	0.87	0.88	0.93	0.05	29281.00

GFI, goodness of fit index; AGFI, adjusted GFI; NFI, normed fit index; CFI, comparative fit index; RMSEA, root mean square error of approximation; AIC, Akaike’s information criterion. 25-items version excludes items that do not load on each factor in Table 1.

Following the original study (Andrade et al., 2014), we also examined the factor structure of the short version of the Psi-Q, excluding two items from each of the six factors. The results show that the factor loadings for the three items did not exceed 0.4 but generally showed good coherence (Supplementary Table S2).

### 3.2. Descriptive statistics and reliability

As a result of the factor analysis, a six-factor Psi-Q structure was employed, and its descriptive statistics and internal reliability for the entire scale and each factor were calculated (Table 3). The results show good internal reliability, *α* = 0.78 ~ 0.94, and good retest reliability, *r* = 0.67 ~ 0.82 (*ps* = 0.00), when retests were conducted 1 week apart.

**Table 3:** Descriptive statistics and retest reliability of the Plymouth Sensory Imagery Questionnaire, compared to United Kingdom data.

	Day-1 data (*N* = 389)			Day-2 data (*N* = 344)				UK data from study 2 of Andrade et al. (2014) (*N* = 209)				
	*M*	SD	*α*	*M*	SD	*r*	*p*	M	SD	*t*	*p*	*d*
Psi-Q	5.29	0.80	0.94	5.14	0.82	0.81	0.00	5.22	0.75	1.00	0.30	0.09
Vision	5.75	0.87	0.78	5.58	0.78	0.69	0.00	5.82	0.65	−1.02	0.31	−0.08
Sound	5.81	0.90	0.84	5.58	0.93	0.71	0.00	5.41	0.98	4.91	0.00	0.43
Smell	4.30	1.22	0.81	4.22	1.17	0.68	0.00	4.60	1.16	−3.01	0.00	−0.25
Taste	5.27	1.07	0.83	5.16	1.05	0.72	0.00	4.78	1.25	4.85	0.00	0.44
Touch	5.29	1.02	0.80	5.20	1.02	0.71	0.00	5.49	0.97	−2.31	0.02	−0.20
Body	5.31	0.98	0.79	5.10	1.00	0.67	0.00	5.23	0.96	1.02	0.31	0.09

Psi-Q, Plymouth Sensory Imagery Questionnaire; M, mean; SD, standard deviation.

### 3.3. Examination of construct validity

To examine construct validity, correlations with other measures were calculated (Table 4).

**Table 4:** Correlations between subfactors of the Plymouth Sensory Imagery Questionnaire and correlations with other scales.

		*M*	SD	Psi-Q	Vision	Sound	Smell	Taste	Touch	Body
Psi-Q		5.29	0.80	–	**0.70**	**0.79**	**0.79**	**0.82**	**0.82**	**0.81**
	Vision	5.75	0.87		–	**0.63**	**0.41**	**0.45**	**0.44**	**0.51**
	Sound	5.81	0.90			–	**0.50**	**0.53**	**0.60**	**0.58**
	Smell	4.30	1.22				–	**0.63**	**0.56**	**0.54**
	Taste	5.27	1.07					–	**0.63**	**0.60**
	Touch	5.29	1.02						–	**0.62**
	Body	5.31	0.98							–
AIQ		3.40	0.70	**0.68**	**0.48**	**0.54**	**0.53**	**0.51**	**0.59**	**0.57**
VOIQ		3.10	0.80	**0.59**	**0.33**	**0.39**	**0.56**	**0.49**	**0.47**	**0.49**
Big Five										
	Extraversion	3.74	1.23	**0.27**	**0.21**	**0.23**	**0.22**	**0.21**	**0.23**	**0.18**
	Neuroticism	4.92	1.25	**−0.13**	−0.08	−0.09	**−0.14**	−0.06	**−0.13**	−0.09
	Openness	4.01	1.05	**0.19**	**0.16**	**0.13**	**0.15**	**0.12**	**0.17**	**0.16**
	Conscientiousness	4.25	1.09	0.10	0.05	0.08	**0.13**	**0.12**	0.07	0.02
	Agreeableness	4.26	1.04	**0.14**	**0.14**	**0.11**	**0.14**	0.08	**0.15**	0.05
FFMQ		3.15	0.38	**0.24**	**0.16**	**0.15**	**0.21**	**0.16**	**0.27**	**0.16**
	Observe	3.47	0.72	**0.32**	**0.21**	**0.24**	**0.28**	**0.18**	**0.30**	**0.28**
	Non-react	2.90	0.67	**0.13**	**0.12**	0.07	**0.11**	0.09	**0.17**	0.05
	Non-judge	2.99	0.65	−0.04	−0.03	−0.01	−0.06	−0.04	−0.02	−0.03
	Describe	2.88	0.73	**0.16**	0.09	0.08	**0.19**	0.09	**0.20**	0.08
	Actaware	3.49	0.72	0.06	0.04	0.00	0.05	0.10	0.07	0.03
SWLS		3.64	1.41	**0.24**	**0.24**	**0.24**	**0.17**	**0.16**	**0.17**	**0.18**
Age		40.72	10.73	**0.21**	**0.10**	**0.16**	**0.28**	**0.17**	**0.15**	**0.12**
Gender		1.60	0.49	**0.14**	**0.12**	**0.18**	0.06	0.08	**0.15**	0.10
Education		3.33	0.95	−0.01	−0.03	−0.05	0.01	0.03	0.03	−0.04

M, mean; SD, standard deviation; Psi-Q, Plymouth Sensory Imagery Questionnaire; AIQ, Auditory Imagery Questionnaire; VOIQ, Vividness of Olfactory Imagery Questionnaire; FFMQ, Five Facet Mindfulness Questionnaire; SWLS, Satisfaction With Life Scale. Bolded figures have a value of *p* of less than 0.05. Gender is numbered 1 for men and 2 for women.

The results show moderate to high correlations and validity with existing measures of imagery ability, with total Psi-Q scores correlating with the AIQ (*r* = 0.68, *p* = 0.00), which measures auditory imagery ability, and the VOIQ (*r* = 0.59, *p* = 0.00), which measures olfactory imagery ability. There are also positive correlations with the Big Five factors of extraversion (*r* = 0.27, *p* = 0.00), openness (*r* = 0.19, *p* = 0.00), and agreeableness (*r* = 0.14, *p* = 0.00), and a negative correlation with neuroticism (*r* = −0.13, *p* = 0.01). In addition to the positive correlation between the total FFMQ scores and the Psi-Q (*r* = 0.24, *ps* = 0.00), the Observe factor and each subfactor of the Psi-Q shows moderate correlations (*r* = 0.18 ~ 0.32, *p* = 0.00). Life satisfaction and total scores on the Psi-Q and each subscale are positively correlated (*r* = 0.16 ~ 0.24, *p* = 0.00). Age (*r* = 0.21, *p* = 0.00) and gender (*r* = 0.14, *p* = 0.01) also correlated positively with the Psi-Q. This indicates that older participants and female participants have higher imagery ability. These correlations indicating construct validity were significant, but the effect sizes were week to moderate.

### 3.4. Cultural comparison

**Figure 1:**
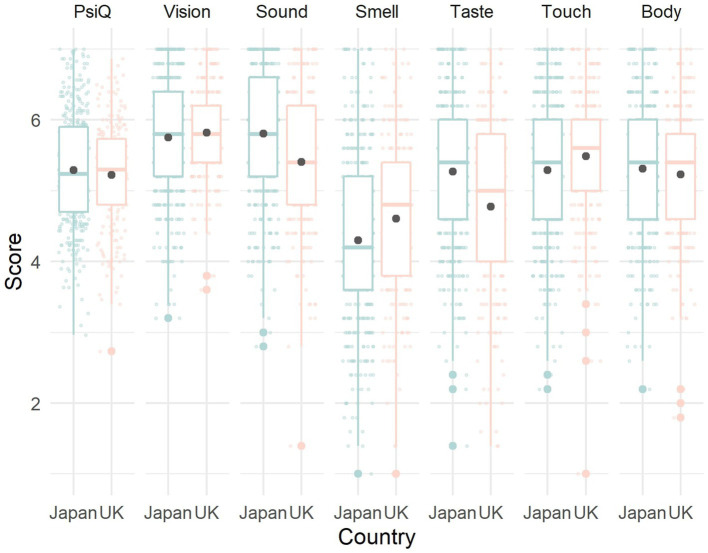
Box plot showing the Japanese-United Kingdom comparison of Plymouth Sensory Imagery Questionnaire.

Finally, we compare the Psi-Q scores of the Japanese sample in this study with the British sample in the original study (Table 1 and Figure 1). ANOVA results showed a non-significant main effect of culture [*F*(1, 596) = 1.06, *p* = 0.30, η^2^ = 0.001], a significant main effect of modality [*F*(6, 3,576) = 243.31, *p* = 0.00, η^2^ = 0.13], and a significant interaction [*F*(6, 3,576) = 28.17, *p* < 0.001, η^2^ = 0.02]. Multiple comparison showed significant differences of sound [*F*(1, 596) = 25.50, *p* < 0.001, η^2^ = 0.04], smell [*F*(1, 596) = 8.81, *p* = 0.003, η^2^ = 0.01], taste [*F*(1, 596) = 25.75, *p* < 0.001, η^2^ = 0.04], and touch [*F*(1, 596) = 5.18, *p* = 0.02, η^2^ = 0.01] between cultures. For sound and taste, Japanese scores were higher than British scores. For smell and taste, British scores were higher than Japanese scores. The culture differences for overall Psi-Q score [*F*(1, 596) = 1.06, *p* = 0.30, η^2^ = 0.002], vision [*F*(1, 596) = 0.88, *p* = 0.35, η^2^ = 0.002], and body [*F*(1, 596) = 1.02, *p* = 0.31, η^2^ = 0.002] were non-significant.

## 4. Discussion

### 4.1. The Japanese version of Plymouth Sensory Imagery Questionnaire

In this study, we developed a Japanese version of the Psi-Q (Andrade et al., 2014), which measures individual differences in multisensory imagery abilities. Internal reliability and retest reliability for this scale were high. The original version measured imagery ability on seven subscales: vision, sound, smell, taste, touch, body, and emotion; however, the Japanese version, which was administered to a Japanese sample, showed the best coherence on six subscales, excluding the emotion factor. When considering the sensory organs, the emotional factor is rarely included, and it is thought to work well as a scale measuring multisensory imagery ability.

### 4.2. Construct validity of the Japanese version of Psi-Q

To examine construct validity, we used scales that have already been shown to be related to existing imagery ability scales and the Psi-Q, as well as scales that are newly examined in the present study. First, the validity of the Japanese version of the Psi-Q was confirmed by its moderate to high positive correlations with olfactory imagery ability (Gilbert et al., 1998) and auditory imagery ability (Hishitani, 2009). In particular, the correlation between olfactory imagery ability, as measured by the VOIQ, and the smell subfactor, measured on the Psi-Q, was higher than the correlations for the other modalities, suggesting that it is correctly measuring what it should measure. We also replicated previous research (Kharlas and Frewen, 2016) and found correlations with trait mindfulness, which has been shown to be related to multisensory imagery ability. Individual differences in mental imagery vividness are thought to be related to actual perceptual, emotional, and physical experiences (Cui et al., 2007; Kharlas and Frewen, 2016). Indeed, a positive correlation between participants’ ages and Psi-Q total and subfactor scores was found in this study and suggests that an increase in the accumulation of real experience makes images more vivid until the middle age of life. In this way, it can be interpreted that mental imagery vividness is related to methods of self-emotional and physical regulation, such as mindfulness. We further found a relationship between multisensory imagery ability and the Big Five personality factors. Specifically, extraversion was associated with imagery vividness in all modalities. Again, this could be explained similarly to mindfulness and age, as individuals with high extraversion are likely to have more sense or opportunity to have a greater variety of perceptual and emotional experiences than those with low extraversion, which may be linked to mental imagery ability. Similarly, in this study, we found, for the first time, a positive correlation between multisensory imagery ability and life satisfaction. Individuals with vivid multisensory imagery may lead more fulfilling lives, both in their everyday and non-everyday life. For example, it is known that the higher their visual imagery ability, the more people appreciate the beauty of poetry (Hitsuwari and Nomura, 2021), which may potentially enhance their art experience. These relationships between trait mindfulness, the Big Five, life satisfaction, and multisensory imagery ability are correlational, and causal relationships are unresolved and should be explored in future research.

### 4.3. Cultural comparison of multisensory imagery ability

This study was the first to examine cultural differences in multisensory imagery ability. The results reveal that while there is no difference in general imagery ability between the Japanese and British samples, however, auditory and gustatory imagery is higher among the Japanese sample, and olfactory imagery is higher among the British sample. By measuring multisensory imagery simultaneously, we can show these differences by modality. First, regarding differences in sound imagery, there are cultural differences in the occurrence of the McGurk effect,^4^ which is less pronounced in the Japanese sample (Sekiyama, 1994). Sekiyama (1994) has argued that Japanese people are less likely to engage in face-to-face communication and the simplicity of the Japanese phonological structure may allow language interaction based on auditory information alone, without reference to visual information. Second, regarding smell imagery, the influence of COVID-19 must be considered (c.f., Olofsson and Pierzchajlo, 2021). In 2022, the year in which data were collected from the Japanese sample, people were permanently wearing masks to prevent the spread of infection; however, this was not so in 2014 when the data were collected from the British sample. This continued wearing of masks (Ataka, 2022) may have caused reduced smell sensitivity and imagery for the Japanese sample. Third, regarding taste imagery, it is thought that there are cultural differences regarding sensitivity to taste. For example, umami is the fifth primary taste in European taste tests and tends to be defined as salty or sweet (Mueller et al., 2011), however, Japanese people are more familiar with umami foods, such as *dashi* (Satoh-Kuriwada et al., 2014). Nevertheless, these interpretations of cultural differences particularly in sound, smell, and taste are only tentative and must be verified in the future. Furthermore, the Psi-Q has already been translated into Spanish (Pérez-Fabello and Campos, 2020) and Dutch (Woelk et al., 2022), and comparative studies with more diverse cultures should be conducted.

### 4.4. Limitation and future direction

Although we were able to develop a Japanese version of the Psi-Q and validate its validity and reliability in this study, several limitations must be mentioned. First, as with the original and translated versions (Andrade et al., 2014; Pérez-Fabello and Campos, 2020; Woelk et al., 2022), the short version was created by reducing items from the long version, and some concerns have been noted with this method of creation (c.f., Aquino et al., 2018), as the same item may be regarded differently in the long and short versions. Second, although we made cultural comparisons, these are exploratory results, and based on the results obtained in this study, hypotheses need to be developed and factors that cause cultural differences in multisensory mental imagery abilities need to be further explored.

## 5. Conclusion

In this study, we developed a Japanese version of the Psi-Q, a scale measuring multisensory imagery ability with high validity and reliability and were able to produce a sufficient measure. Additionally, correlations with various scales, including the Big Five and life satisfaction scales, which had not been examined before, were clarified, and the adequate construct validity was demonstrated. In the Japanese-British comparison, cultural differences in multisensory imagery ability between the Japanese and British cultures could be noted for the first time. In the future, comparing the Psi-Q with other scales and using it in combination with behavioral experiments and functional brain imaging studies can increase our understanding of multisensory imagery ability. Future research should also conduct multicultural comparisons, not only between Japanese and British samples. The number of studies dealing with multiple modality senses simultaneously remains small, and it is expected that the Psi-Q will enable us to advance this field.

## Data availability statement

The datasets presented in this study can be found in online repositories. The names of the repository/repositories and accession number(s) can be found in the article/Supplementary material.

## Ethics statement

The studies involving human participants were reviewed and approved by Ethics Committee of Kyoto University. The patients/participants provided their written informed consent to participate in this study.

## Author contributions

JH: conceptualization, methodology, software, formal analysis, writing – original draft, and visualization. MN: conceptualization, methodology, writing – review and editing, and supervision. All authors contributed to the article and approved the submitted version.

## Funding

This research was supported by Grants-In-Aid for Scientific Research (JSPS KAKENHI; Grant Number: 20K20863 and 22H01103).

## Conflict of interest

The authors declare that the research was conducted in the absence of any commercial or financial relationships that could be construed as a potential conflict of interest.

## Publisher’s note

All claims expressed in this article are solely those of the authors and do not necessarily represent those of their affiliated organizations, or those of the publisher, the editors and the reviewers. Any product that may be evaluated in this article, or claim that may be made by its manufacturer, is not guaranteed or endorsed by the publisher.

## Footnotes

^1^ https://osf.io/u8cpd/

^2^ https://crowdworks.jp/

^3^ https://www.nai.co.jp/

^4^A phenomenon in which a third phoneme is perceived when a video of one phonological speech utterance is viewed in combination with another (McGurk and MacDonald, 1976).
