# Supplementary material for: Developing and validating a Japanese version of the Plymouth Sensory Imagery Questionnaire
Source: Front Psychol. 2023 Jun 23;14:1166543. doi: 10.3389/fpsyg.2023.1166543 (PMC10327475; doi:10.3389/fpsyg.2023.1166543)
Supplement: Supplementary file 1 [file Table_1.docx]

**Supplementary Table 1.**

Factor loadings for each item in the factor analysis assuming 8-factor structure (only items with factor loadings of 0.4 or higher are shown)

|  |  | MR7 | MR5 | MR3 | MR1 | MR2 | MR4 | MR8 | MR6 |
| --- | --- | --- | --- | --- | --- | --- | --- | --- | --- |
| Imagine the appearance of… | |  |  |  |  |  |  |  |  |
| 1 | *a bonfire |  |  |  |  | .74 |  |  |  |
| 2 | *a sunset |  |  |  |  | .66 |  |  |  |
| 3 | *a cat climbing a tree |  |  |  |  | .52 |  |  |  |
| 4 | a friend you know well |  |  |  |  | .48 |  |  |  |
| 5 | the front door of your house |  |  |  |  |  |  |  |  |
| Imagine the sound of… | |  |  |  |  |  |  |  |  |
| 6 | *the sound of a car horn | .64 |  |  |  |  |  |  |  |
| 7 | *hands clapping in applause | .43 |  |  |  |  |  |  |  |
| 8 | *an ambulance siren. | .72 |  |  |  |  |  |  |  |
| 9 | the sound of children playing | .55 |  |  |  |  |  |  |  |
| 10 | the mewing of a cat | .53 |  |  |  |  |  |  |  |
| Imagine the smell of… | |  |  |  |  |  |  |  |  |
| 11 | *newly cut grass |  |  |  |  |  |  |  |  |
| 12 | *burning wood |  |  |  |  |  | .67 |  |  |
| 13 | *a rose |  |  |  |  |  | .52 |  |  |
| 14 | fresh paint |  |  |  |  |  | .58 |  |  |
| 15 | a stuffy room |  |  |  |  |  | .44 |  |  |
| Imagine the taste of | |  |  |  |  |  |  |  |  |
| 16 | *black pepper |  | .63 |  |  |  |  |  |  |
| 17 | *lemon |  | .57 |  |  |  |  |  |  |
| 18 | *mustard |  | .68 |  |  |  |  |  |  |
| 19 | toothpaste |  | .51 |  |  |  |  |  |  |
| 20 | sea water |  |  |  |  |  |  |  |  |
| Imagine touching… | |  |  |  |  |  |  |  |  |
| 21 | *fur |  |  |  | .48 |  |  |  |  |
| 22 | *warm sand |  |  |  | .61 |  |  |  |  |
| 23 | *a soft towel |  |  |  |  |  |  |  |  |
| 24 | icy water |  |  |  | .41 |  |  |  |  |
| 25 | the point of a pin |  |  |  | .48 |  |  |  |  |
| Imagine the bodily sensation of | |  |  |  |  |  |  |  |  |
| 26 | *relaxing in a warm bath |  |  |  |  |  |  | .69 |  |
| 27 | *walking briskly in the cold |  |  |  |  |  |  |  |  |
| 28 | *jumping into a swimming pool |  |  |  |  |  |  |  |  |
| 29 | having a sore throat |  |  |  |  |  |  | .42 |  |
| 30 | threading a needle |  |  |  |  |  |  |  |  |
| Imagine feeling | |  |  |  |  |  |  |  |  |
| 31 | *excited |  |  | .72 |  |  |  |  |  |
| 32 | *relieved |  |  |  |  |  |  |  | .60 |
| 33 | *scared |  |  | .82 |  |  |  |  |  |
| 34 | furious |  |  | .76 |  |  |  |  |  |
| 35 | in love |  |  |  |  |  |  |  | .80 |

**Supplementary Table 2.**

Factor loadings for each item in the factor analysis assuming 6-factor structure

|  |  | MR6 | MR4 | MR1 | MR5 | MR2 | MR3 | h2 | u2 |
| --- | --- | --- | --- | --- | --- | --- | --- | --- | --- |
| Imagine the appearance of… | |  |  |  |  |  |  |  |  |
| 1 | *a bonfire | .02 | -.04 | .00 | .02 | .75 | .10 | .62 | .38 |
| 2 | *a sunset | .03 | .09 | .07 | .06 | .69 | -.09 | .61 | .39 |
| 3 | *a cat climbing a tree | .20 | -.03 | .00 | .03 | .52 | .13 | .50 | .50 |
| 4 | a friend you know well | .02 | .13 | -.06 | .11 | .51 | -.07 | .35 | .65 |
| 5 | the front door of your house | .29 | .10 | -.07 | -.03 | .24 | -.08 | .21 | .79 |
| Imagine the sound of… | |  |  |  |  |  |  |  |  |
| 6 | *the sound of a car horn | .70 | -.04 | .00 | .09 | .03 | .05 | .58 | .42 |
| 7 | *hands clapping in applause | .45 | -.08 | .30 | -.07 | .21 | .05 | .54 | .46 |
| 8 | *an ambulance siren. | .74 | .01 | .00 | -.03 | .01 | -.02 | .54 | .46 |
| 9 | the sound of children playing | .57 | .10 | .06 | .06 | -.06 | .07 | .47 | .53 |
| 10 | the mewing of a cat | .57 | .03 | -.02 | .07 | .21 | .01 | .57 | .43 |
| Imagine the smell of… | |  |  |  |  |  |  |  |  |
| 11 | *newly cut grass | .20 | .10 | .28 | .05 | -.11 | .31 | .41 | .59 |
| 12 | *burning wood | .01 | .07 | .06 | .04 | .17 | .64 | .59 | .41 |
| 13 | *a rose | .10 | .30 | -.03 | -.04 | .09 | .49 | .49 | .51 |
| 14 | fresh paint | .15 | .17 | .12 | .08 | -.08 | .52 | .53 | .47 |
| 15 | a stuffy room | .02 | .07 | .17 | .30 | -.05 | .38 | .44 | .56 |
| Imagine the taste of | |  |  |  |  |  |  |  |  |
| 16 | *black pepper | .00 | .68 | .01 | .10 | -.01 | .12 | .61 | .39 |
| 17 | *lemon | .03 | .57 | .25 | -.04 | .15 | -.15 | .55 | .45 |
| 18 | *mustard | -.01 | .74 | -.01 | -.01 | .04 | .17 | .66 | .34 |
| 19 | toothpaste | .19 | .51 | .00 | .23 | -.02 | -.16 | .53 | .47 |
| 20 | sea water | -.03 | .30 | .31 | .08 | .08 | .17 | .46 | .54 |
| Imagine touching… | |  |  |  |  |  |  |  |  |
| 21 | *fur | .12 | .06 | .52 | -.01 | .03 | .01 | .40 | .60 |
| 22 | *warm sand | -.04 | .00 | .64 | .09 | .08 | .09 | .55 | .45 |
| 23 | *a soft towel | .16 | .17 | .37 | .20 | .05 | -.12 | .52 | .48 |
| 24 | icy water | .19 | .18 | .45 | .19 | .02 | -.18 | .61 | .39 |
| 25 | the point of a pin | .06 | -.02 | .53 | .03 | -.06 | .12 | .35 | .65 |
| Imagine the bodily sensation of | |  |  |  |  |  |  |  |  |
| 26 | *relaxing in a warm bath | .09 | -.01 | -.06 | .68 | .10 | -.05 | .54 | .46 |
| 27 | *walking briskly in the cold | -.03 | -.02 | .14 | .46 | .10 | .18 | .40 | .60 |
| 28 | *jumping into a swimming pool | -.02 | -.06 | .16 | .48 | .16 | .16 | .45 | .55 |
| 29 | having a sore throat | .00 | .10 | .08 | .55 | .03 | -.09 | .42 | .58 |
| 30 | threading a needle | .11 | .11 | .05 | .50 | -.07 | .11 | .45 | .55 |
